# The difference between two brachycephalic and one mesocephalic dog breeds’ problem-solving performance suggests evidence for paedomorphism in behaviour

**DOI:** 10.1038/s41598-023-41229-8

**Published:** 2023-09-21

**Authors:** Dorottya Júlia Ujfalussy, Zsófia Bognár, Marianna Molnár, Ádám Miklósi, Enikő Kubinyi

**Affiliations:** 1 Department of Ethology, Institute of Biology, ELTE Eötvös Loránd University, Pázmány Péter sétány 1/C, Budapest, 1117 Hungary; 2 MTA-ELTE Lendület “Momentum” Companion Animal Research Group, Budapest, Hungary; 3 Faculty of Natural Sciences, Centre for Environmental Research, Eötvös Loránd University, Budapest, Hungary; 4 ELTE NAP Canine Brain Research Group, Budapest, Hungary; 5 ELTE-ELKH NAP Comparative Ethology Research Group, Institute of Cognitive Neuroscience and Psychology, Research Centre for Natural Sciences, Budapest, Hungary

**Keywords:** Evolution, Neuroscience, Psychology, Structural biology

## Abstract

Despite serious health and longevity problems, small brachycephalic breeds are becoming increasingly popular among pet owners. Motivations for choosing short-nosed breeds have been extensively investigated in recent years; however, this issue has been addressed mainly by relying on owner reports, resulting in explanations of “cute looks”, referring to the baby-schema phenomenon and “behaviour well suited for companionship”. We aimed to compare the behaviour of two brachycephalic (English and French bulldogs) and one mesocephalic (Mudi) breed in a problem-solving context. The dogs were given the task of opening boxes containing food rewards. We investigated human-directed behaviour elements over success and latency (indicators of motivation and ability). We found that both English and French bulldogs were significantly less successful in solving the problem than mudis. Both brachycephalic breeds had longer opening latencies than the mesocephalic breed. Brachycephalic breeds oriented less at the problem box and more at humans present. In summary, the short-headed breeds were less successful but oriented much more toward humans than mesocephalic dogs. Owners might interpret these behaviours as “helplessness” and dependence. The results support the hypothesis that infant-like traits may be present not only in appearance but also in behaviour in brachycephalic breeds, eliciting caring behaviour in owners.

## Introduction

Short-headed, flat-faced, small companion dog breeds (so-called small brachycephalic breeds) are becoming increasingly popular among dog owners^1–5^, even though brachycephalism is associated with a range of serious health and welfare issues^3,6–8^. This puzzling phenomenon could be described as the “Flat-Faced Paradox”. Despite the efforts to raise awareness of future owners, currently, the French bulldog is the second most popular breed in the UK and the USA^1,9^ and first in Hungary^10^, while other flat-faced breeds are also among the top popular breeds, e.g. the English bulldog, the Pug, the Boston terrier and the Shih Tzu. Brachycephalic breeds tend to suffer from breathing problems (such as brachycephalic obstructive airway syndrome—BOAS e.g.^11^), eye diseases e.g.^12^, spinal malformations/neurological issues e.g.^13,14^, brain disorders^3^, sleeping disorders e.g.^15^, skin^3^, ear and dental diseases^3^, gastrointestinal disorders e.g.^16,17^, thermoregulation problems e.g.^18^, exercise intolerance^6^, and above all, have difficulties giving birth naturally e.g.^19^. Furthermore, mostly because of their health problems, small brachycephalic breeds have a considerably shorter expected lifespan than their longer-headed counterparts^3,20,21^. Nonetheless, some owners still are highly likely to reacquire (93%) these breeds and recommend them to prospective owners (65.5%)^22^. High levels of health problems can even positively affect the owner-dog attachment^23^.

The main reasons reported by owners are that these breeds are supposedly suitable for households with children, well suited for a sedentary lifestyle and display positive behaviour traits for companionship, the latter being a key factor^24^. While no evidence has been found that these breeds have a lower risk factor for biting children^25^, no doubt that they are suitable for a sedentary lifestyle, as they have been shown to suffer from excessive exercise intolerance^26,27^. However, the reported “positive behaviour traits for companionship” are worth further investigation, as various interesting behavioural differences between brachycephalic and non-brachycephalic breeds have been identified. For example, dogs with higher cephalic indexes (brachycephalic or “short” headed) have been found to be more successful in following the human pointing gesture than dogs with lower cephalic indices^28^. Brachycephalic breeds display longer looking times at human and dog portraits compared to longer-headed breeds^29^. Shorter-headed dogs also have been shown to establish eye contact with humans more readily than longer-headed dogs^30^. Moreover, positive behaviours directed toward unfamiliar humans, such as being affectionate, cooperative and interactive, are associated with a higher cephalic index^31,32^. These findings support the hypothesis that not only the "cute", paedomorphic appearance^33^ but behaviour characteristics (due to genetic predisposition or the result of different treatment) indeed might influence the choice of this breed and the development of breed loyalty.

Paedomorphic (baby-like) appearance, in general, triggers a nurturing, caring instinct across species (baby-schema effect) see e.g.^34–36^. Paedomorphic facial attributes have been found to enhance the chance of adoption in both cats^37^ and dogs^38^. Owners also infer the personality of dogs and expected relationship quality based on physical appearance^39^.

However, baby-like traits may also be present in behaviour. Dogs, in general, display such behaviours that elicit care^40–42^, but breeds with a more baby-like appearance could also be more prone to display “helpless”, baby-like, care-inducing behaviours compared to breeds with a longer head shape.

In an unsolvable task paradigm, dogs, but not wolves, have been found to look back at humans^43^, which has been widely interpreted as a communicative, perhaps even “assistance seeking” intent (but also see^44^), or as an indicator of generalised dependence on human action^45^. Whichever the case, looking back at the human in an independent problem-solving task is most probably interpreted by the human counterpart as help-seeking or at least a sign of helplessness, which may trigger the nurturing instinct. While differences in looking back behaviour in an independent problem-solving context may be mediated by various factors, such as life experience, training history^46–49^ or anxiety-related disorders^50^, we suggest that such context may still be suitable to study differences in care-inducing behaviours.

In this study, we aimed to compare two brachycephalic and one mesocephalic breeds' behaviour in three similar independent problem-solving tasks, slightly differing in difficulty. We not only wanted to compare success rates but also, more importantly, wished to check for differences in human-directed behaviour and thus search for evidence of paedomorphic behaviour traits, which may be appealing to present and prospective owners.

## Method

### Subjects

We assessed 15 English bulldogs [10 males, 5 females, age range 0.75–9.92 years (average. 3.71 years)], 15 French bulldogs [5 males, 10 females, age range 0.67–7.00 years (average 2.34 years)], and 13 Hungarian mudis [7 males, 6 females, age range 1.50–11.00 years (average 4.29 years)] in behaviour tests. All subjects from all three breeds were recruited similarly. Owners were approached on annual meetings of the breed (so called Breed Meet Days), where they were asked if they are willing to volunteer for a behaviour test. If they agreed to do so, they were informed about the procedure and asked to sign a consent form before the experiment started. 

### Equipment

**Figure 1:**
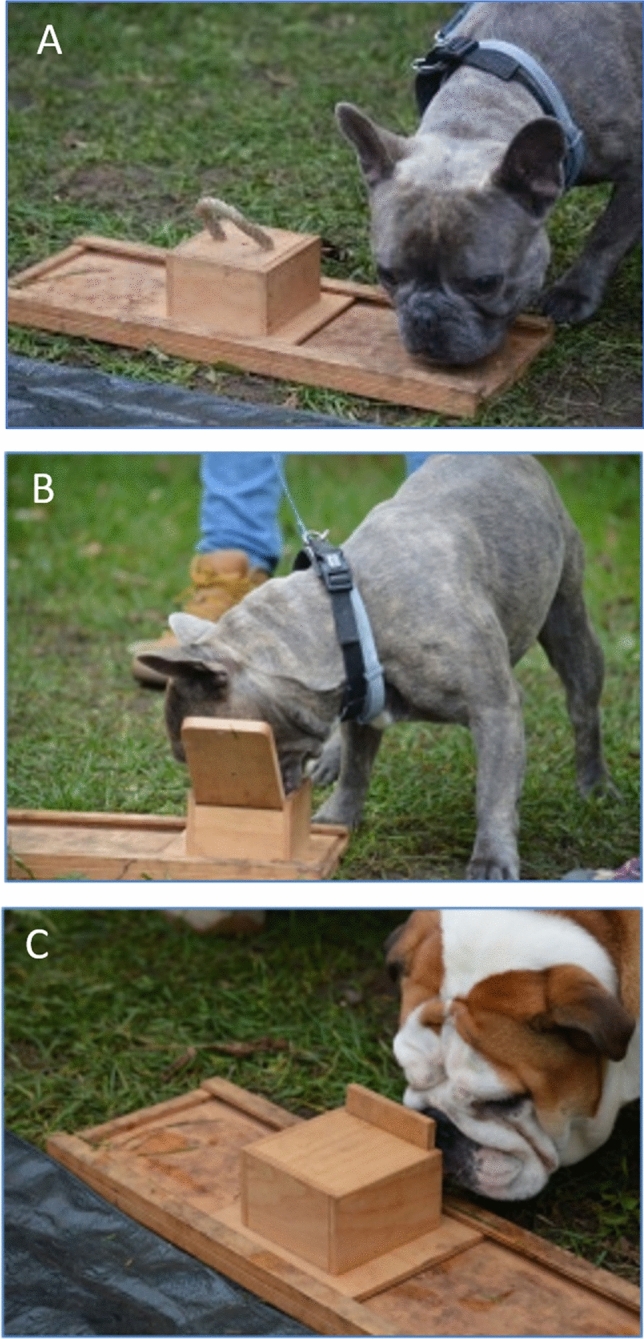
Illustration of the experiment—problem boxes (**A**–**C**).

We have used three commercially available problem boxes intended for dogs (Games4Brain Dog Intelligence Game Set), made of plywood, all mountable to a 50 cm long plywood rail to prevent knocking over. All boxes had a different opening technique, growing in difficulty, with Box A being the most difficult to open and Box C being the easiest. Please see Fig. 1 for the pictures of the boxes and the opening technique.

### Procedure

The testing in case of all three breeds was carried out during breed meetings at an outdoor location. The dogs had an opportunity to familiarize with the location prior to testing. The testing site was separated by at least 30 m from all other activity. The dog was facing the problem box, held loosely on a leash by the Owner, who was standing behind the dog slightly shifted to one side. The experimenter was initially standing in front of the dog, on the opposite side of the problem box. All trials started with the Experimenter drawing the dog’s attention to the reward (a small piece of Viener sausage), then opening the box and placing the reward inside, while being observed by the dog, still held on a leash by the Owner, approx. 1 m from the box. After the Experimenter visibly placed the reward into the box and showed an empty hand, she moved behind the dog and stood beside the Owner. When this position was taken up (both humans were stituated behind the dog’s line of site), the dog was allowed to attempt to open the box. Visual contact between dogs and humans was only restricted by their position. Dogs were allowed to try to open the problem boxes for 2 min. The boxes were presented once each, in a random order, with a 3 min. the interval between trials, three trials in total. All sessions were video recorded.

### Data analysis

Videos of testing trials were coded for success and latencies to open the problem box, as well as for behaviour variables in orientation of the face, nose use and paw use. The complete list of coded variables may be found in Table 1.

**Table 1:** Coded variables.

Variable	Definition	Measure
Opening success	Whether the subject succeeded in opening the box to obtain the reward within the 120-s timeframe of the trial	Yes (1) No (0)
Opening latency	The time it took to open the box and obtain the reward	s
Orienting^a^ at Owner	Amount of time spent orienting toward the Owner	s
Orienting^a^ at Experimenter	Amount of time spent orienting toward the Experimenter	s
Orienting^a^ at box	Amount of time spent orienting toward the target box	s
Nose usage	Total time of nose/muzzle touching the target box	s
Paw usage	Total time of any of the paws touching the target box	s

^a^Orienting defined as the direction the subjects face is turning.

We analysed the data using R statistical software (version 4.1.1)^51^ in Rstudio^52^.

The time measures of Orienting at the Owner, Orienting at the Experimenter, and Orienting at the box, were transformed into percentage of the total time because the length of the trials depended on the Opening latency. The time measures of Nose usage and Paw usage were transformed to the percentage of the manipulation time. All subjects manipulated the box at least once. Due to the mutually exclusive connection found between Nose usage and Paw usage, the large proportion of Nose usage (89.52 ± 24.47% of manipulation time) and the rarity of Paw usage (10.48 ± 24.47% of manipulation time), this data was transformed to Manipulation type binary score (0: only nose usage, 1: paw usage occurs) later. As the orientations (owner, experimenter, box) were closely connected, we ran a principal component analysis to summarise the behaviour variables into summary indices (“principal” functions of “psych” package^53^), after using parallel analysis to determine the number of components to extract (“fa. parallel” function of “psych” package^53^). All the behaviour variables were loaded into one component: the social behaviours (orienting toward the owner/experimenter) positively, and the non-social behaviours (orienting toward the box) negatively (Table 2). Thus, the component represented a social strategy over a problem-oriented strategy. Therefore, we named it ‘social strategy score’ (similarly to^54^). After calculating the social strategy score, we checked the internal consistency of the factor with Cronbach’s alpha (“alpha” function of “psych” package^53^).

**Table 2:** Factor loadings of principal component analysis.

Behaviour variables	– Min to max	Mean ± SD	Factor loadings (social strategy score)
Percentage of time spent orienting toward the Owner	0 to 36.50%	7.39 ± 9.70%	0.782
Percentage of time spent orienting toward the Experimenter	0 to 53.43%	15.96 ± 15.96%	0.806
Percentage of time spent orienting toward the target box	1.50 to 100%	58.27 ± 32.79%	− 0.948
			SS loadings: 2.162
			Proportion Var: 0.721
			Cronbach’s alpha: 0.801

Binomial Generalised Linear Mixed Model with logit link (“glmer” function of “lme4” package^55^) was used to check the possible associations between the opening success and dogs’ (1) breed, (2) box type and (3) sex as fixed factors, and (4) age as a covariate, and (5) manipulation type as binary score, with subject ID as a random factor.

Survival analysis was suggested for latency outcomes in behavioural experiments^56^; thus we used the Mixed Effects Cox Regression Model (“coxme” function of “coxme” package^57^) to analyse the effect of (1) breed, (2) box type and (3) sex as fixed factors, and (4) age as covariate, and (5) manipulation type as a binary score on the opening latency, with subject ID as a random factor.

Due to the distribution of the orientation factor score data (social strategy score), Zero-Inflated Beta Regression Mixed Model (“glmmTMB” function of “glmmTMB” package^58^) was used to examine its possible associations with (1) breed, (2) box type and (3) sex as fixed factors, and (4) age as a covariate, with subject ID as a random factor.

A Binomial Generalised Linear Mixed Model with logit link (“glmer” function of “lme4” package^55^) was used to check the possible associations between manipulation type and (1) breed, (2) box type and (3) sex as fixed factors, and (4) age as a covariate, with subject ID as a random factor.

For all the models, bottom-up model selection was used (“anova” function of “stats” package^51^), where the inclusion criteria were a significant likelihood ratio test for each tested variable. The most parsimonious models are presented below. A Tukey post-hoc test was used for comparisons between the three breed groups and the three box types (“emmeans” function of “emmeans” package^59^).

### Ethical statement

The reported research project is in accordance with the Ethical Guidelines of Research in Hungary. The behaviour experiment was conducted with the permission of the National Animal Experimentation Ethics Committee (number of ethical permission: PE/EA/128-2/2017). Owners volunteered with their dogs to participate in the study, received no monetary compensation and gave written consent. All owners signed an informed consent form. Dogs and owners could freely decide to leave the sessions at any time. Methods are also in agreement with ASAB guidelines.

## Results

### Success

We found a significant effect of breed (p < 0.001) and box type (p < 0.001) on the success, but sex, age or manipulation type effect and no interaction between breed and box type were found. English bulldogs and French bulldogs were less successful than mudis (EB: ß ± SE: − 2.61 ± 0.83; Z = − 3.16; OR = 0.07 [0.01–0.51]; p = 0.005; FB: ß ± SE: − 2.73 ± 0.83; Z = − 3.31; OR = 0.07 [0.01–0.45]; p = 0.003). Dogs were less successful with opening box A and B than box C (A: ß ± SE: − 2.32 ± 0.81; Z = − 2.86; OR = 0.10 [0.01–0.66]; p = 0.012; B: ß ± SE: − 3.17 ± 0.81; Z = − 3.90; OR = 0.04 [0.01–0.28]; p < 0.001).

### Opening latencies

**Figure 2:**
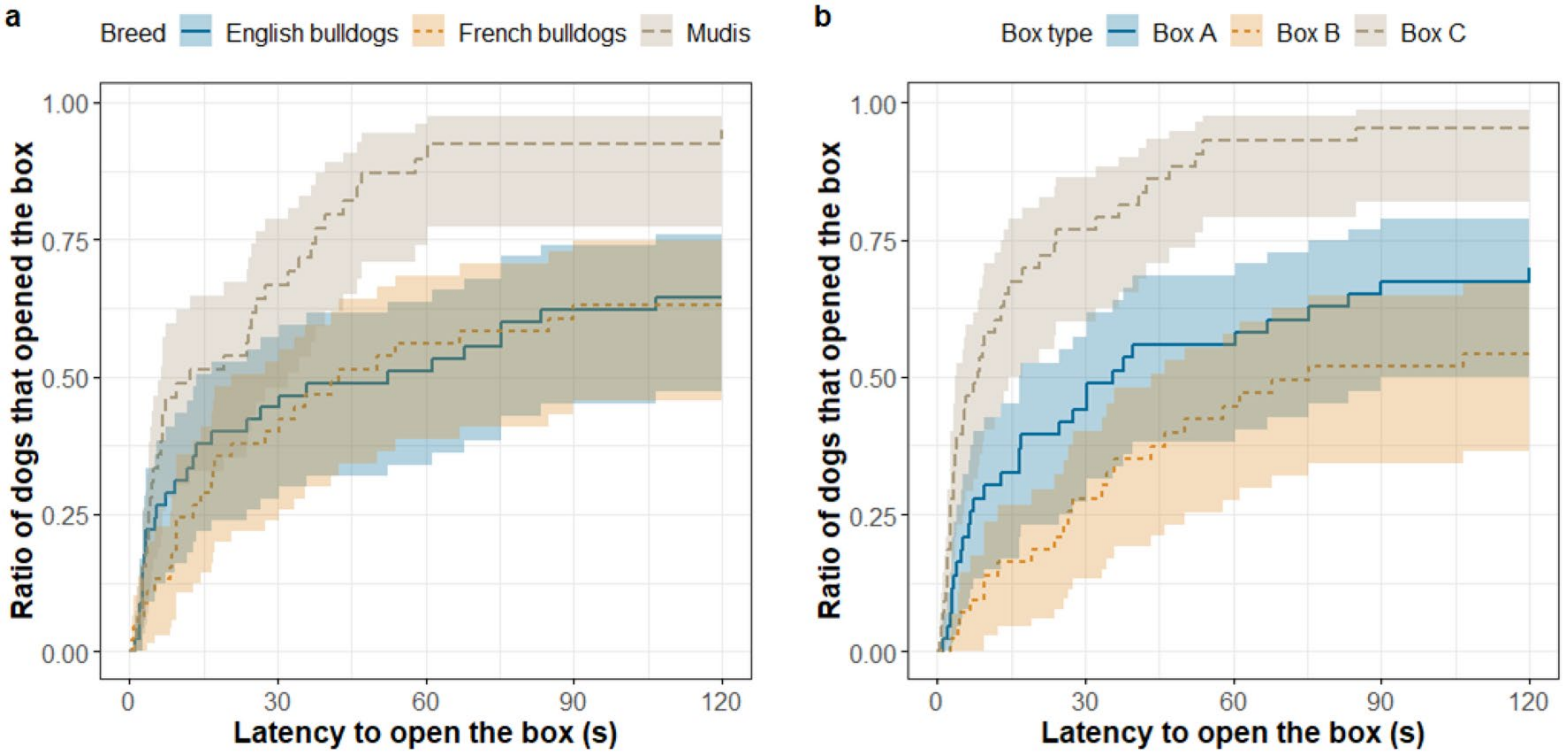
Association between the opening latencies and the three breeds (**a**) and the three box types (**b**)—e.g. after 60 s elapsed, usually ~ 90% of mudis have already opened the box, while at the same time, only ~ 50% of English and French bulldogs had. Also, after 60 s elapsed, usually ~ 90% of dogs have already opened box C, while at the same time, only ~ 55% of dogs opened box A and ~ 45% of dogs opened box B.

We found a significant effect of breed (p = 0.003) and box type (p < 0.001) on the opening latencies, but no sex, age or manipulation type effect and no interaction between breed and box type were found. English bulldogs and French bulldogs were slower than mudis (EB: ß ± SE: − 0.83 ± 0.30; Z = − 2.76; OR = 0.44 [0.22–0.88]; p = 0.016; FB: ß ± SE: − 0.97 ± 0.30; Z = − 3.26; OR = 0.38 [0.19–0.76]; p = 0.003; Fig. 2a). Dogs were slower with opening box A and B than box C (A: ß ± SE: − 1.08 ± 0.26; Z = − 4.22; OR = 0.34 [0.19–0.62]; p < 0.001; B: ß ± SE: − 1.59 ± 0.28; Z = − 5.76; OR = 0.20 [0.11–0.39]; p < 0.001; Fig. 2b).

### Social strategy score

**Figure 3:**
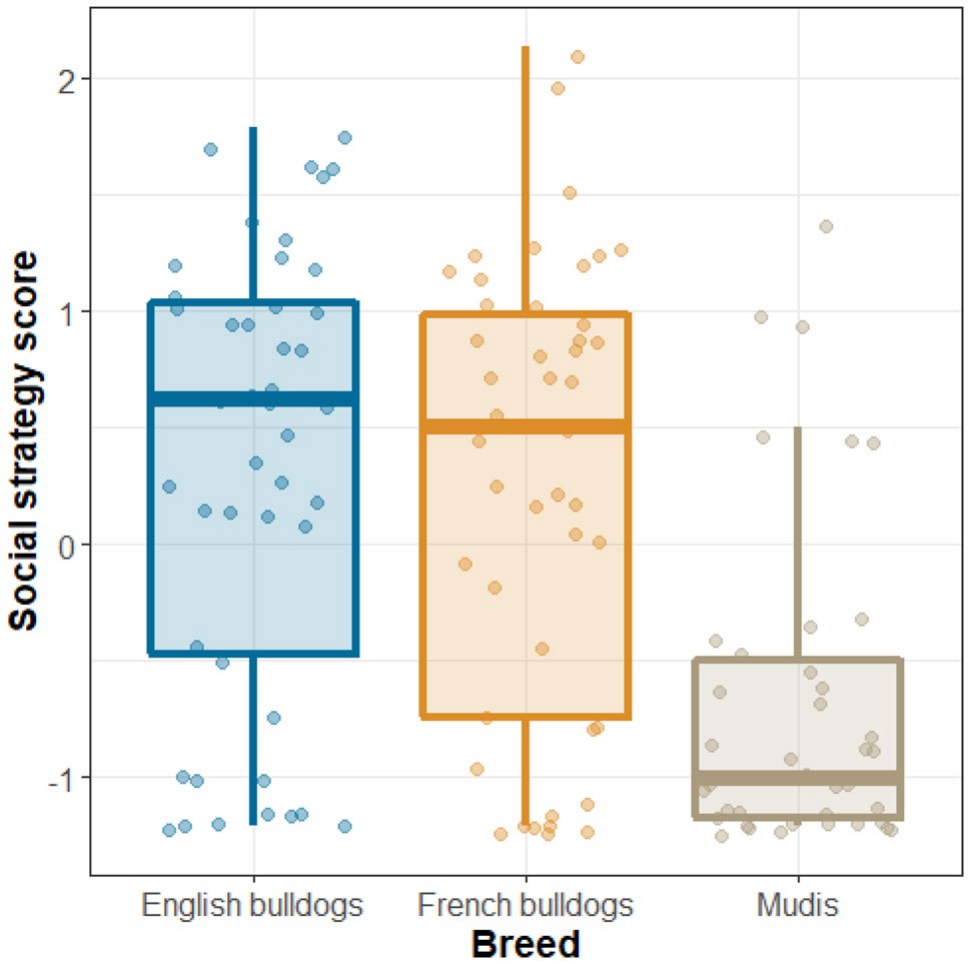
Association between the social strategy score and the three breeds—English and French bulldogs had higher scores (oriented toward the humans more) than mudis.

We found a significant effect of breed (p < 0.001) on the social strategy score, but no box type, sex or age effects were found. English bulldogs and French bulldogs had higher social strategy scores (look back longer at humans) than mudis (EB: ß ± SE: 1.43 ± 0.32; Z = 4.45; OR = 4.16 [1.95–8.91]; p < 0.001; FB: ß ± SE: 1.50 ± 0.32; Z = 4.70; OR = 4.49 [2.10–9.58]; p < 0.001; Fig. 3).

### Manipulation type

**Figure 4:**
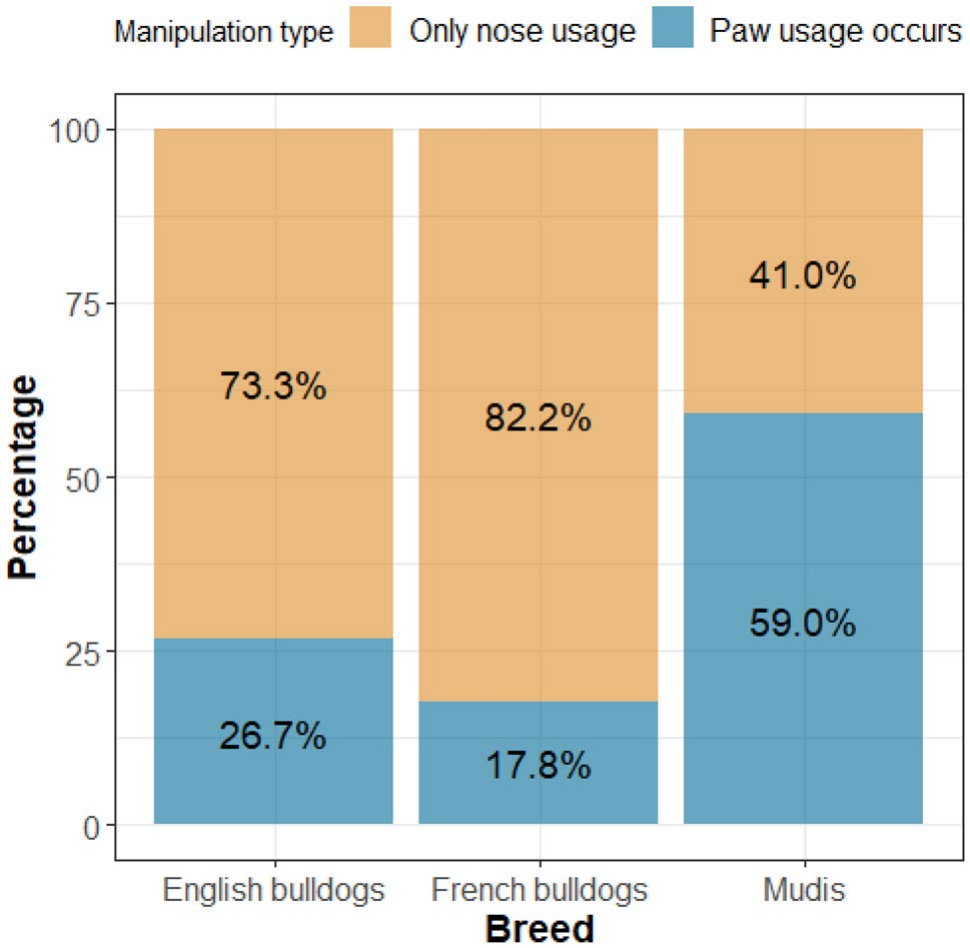
Association between the manipulation type and the three breeds—English and French bulldogs used their paws less than mudis.

We found a significant effect of breed (p < 0.001) and box type (p < 0.001) on manipulation type, but no sex or age effect and no interaction between breed and box type were found. Mudis used their paws more than English bulldogs and French bulldogs (EB: ß ± SE: 1.72 ± 0.54; Z = 3.17; OR = 5.60 [1.56–20.04]; p = 0.004; FB: ß ± SE: 2.35 ± 0.59; Z = 3.95; OR = 10.46 [2.60–42.03]; p < 0.001; Fig. 4). Dogs used their paws more while opening box B than boxes A and C (A: ß ± SE: 1.72 ± 0.54; Z = 3.21; OR = 5.60 [1.59–19.71]; p = 0.004; C: ß ± SE: 2.40 ± 0.60; Z = 4.02; OR = 11.04 [2.72–44.82]; p < 0.001).

## Discussion

In this study, we aimed to identify breed-specific behaviour differences between two brachy- and one mesocephalic breed in a problem-solving context to establish research data corresponding to owner reports of “positive behaviour traits for companionship”^24^. As prospective and present owners often justify their breed choice with this argument, we hypothesised that such differences exist and could be studied in an independent problem-solving context in the presence of owners. Our results seem to corroborate this hypothesis, as brachycephalic dogs orient significantly more at humans present than individuals of a mesocephalic breed when faced with a problem situation. As said before, looking back at the human in a problem situation may be mediated by other factors, such as breed type, life experience^47–49^ anxiety-related disorders^46,50^, as well as some anatomical characteristics which is an undoubted limitation of this study. For example, retinal ganglion cell distribution, and most importantly peak ganglion cell density in the area centralis have been found to be negatively correlated with skull length and positively correlated with cephalic index^60^. This has been suggested to result in better visual focus in the center of the visual field in case of brachycephalic breeds, possibly contributing to better visual communicative abilities in these breeds^28–30^. Moreover, due to brachycephalic dogs’ nostril stenosis^61^, nasal turbinate hypertrophy^62^, and significantly decreased olfactory brain^63^, being “short-nosed” may intuitively suggest poorer olfaction abilities, possibly resulting in lower motivation. However based on results comparing olfaction of brachycephalic and other breeds^64,65^, we may assume that—contrary to expectations—brachycephalic breeds do not differ from the general dog population in their olfaction abilities.

Still, this finding is particularly interesting as communication initiation, such as looking at the owner, was found to be in direct positive correlation with the emotional importance of the dog to the owner, as well as in direct positive correlation with the time spent in active engagement with the dog (Bognár et al. in prep). This suggests that this behaviour may indeed be partly responsible for owners’ desire for short-headed breeds.

Looking at the owner or the experimenter in a problem-solving situation, however, may be enhanced by various factors. On the one hand, small short-headed companion dogs could be genetically more inclined to act less independently (i.e. “baby-like” or “helpless”) to elicit human care. Such a trait would have a distinct advantage in an artificial selection context. On the other hand, it is possible that, mainly due to their physical appearance, owners treat brachycephalic dogs more like children; thus, they get more experience and positive feedback in communicative (“help-seeking”) situations, which experience then causes differences in behaviour. This hypothesis could be tested by contrasting the communicative behaviour of the owners of brachycephalic and mesocephalic dogs in a teaching or helping the situation with their relatively young dogs, possibly revealing more “parent-like” behaviour in owners of short-headed dogs. Also, testing puppies still at breeders in an identical paradigm could help disentangle genetic predispositions and the effect of life experience.

It is also plausible that brachycephalic dogs are less able to solve the problem due to physical constraints. We found evidence for this in limitations in paw use, but interestingly not in nose use. Based on the short nose, not very much protruding from the plane of the face, a natural hypothesis would be that nose use would be compromised in brachycephalic breeds. Our findings, however do not corroborate this hypothesis. Short-headed subjects used their paw less than subjects of the mesocephalic breed, but no difference was detected in case of nose use. A possible explanation of this could be that other anatomical issues, such as for example shorter limbs and neck, shape and weight of the head, rounder paws and body weight being centred over the front limbs^66^, making those more difficult to lift without losing balance, may outweigh the difficulty of manipulations with a short nose. Also, it is worth noting that manipulations by nose and mouth cannot be avoided, as those are absolutely necessary for feeding, while this is not necessarily the case for manipulations with the paws. Unfortunately, we are not able to untangle these factors based on present research. Most probably, it is safe to assume that there is an interaction of all the above-mentioned factors contributing to behaviour differences.

However, we have found stable evidence for enhanced orientation at humans in short-headed dogs when faced with a problem, which the human counterpart may well interpret as “helplessness”, help-seeking, and communication initiation—probably, together with baby-like looks, are a trigger for our basic nurturing instinct.

This may be the main reason why health and welfare-related issues if known, are mostly disregarded by owners acquiring small brachycephalic companion breeds. In their intriguing study on motives underlying pet ownership, Beverland et al.^67^ have found that some owners are even drawn to poor health status and related helplessness and vulnerability, as this makes them feel more needed.

While such motivation is marginal and far from being the standard, it seems that, like in many other instances, humans find it very difficult to cognitively override strong instinctive predispositions and still choose brachycephalic breeds, disregarding future health and welfare issues.

### Supplementary Information

Supplementary Information.

## Data Availability

Raw data is straight forward and not extensive and as such is provided as Supplementary material.
