# Supplementary material for: The difference between two brachycephalic and one mesocephalic dog breeds’ problem-solving performance suggests evidence for paedomorphism in behaviour
Source: Sci Rep. 2023 Sep 21;13:14284. doi: 10.1038/s41598-023-41229-8 (PMC10514333; doi:10.1038/s41598-023-41229-8)
Supplement: Supplementary file 1 — Supplementary Information. [file 41598_2023_41229_MOESM1_ESM.pdf]

Supplementary material for:

The difference between two brachycephalic and one mesocephalic dog breeds' problem-solving performance suggests evidence for paedomorphism in behaviour

Authors:

Dorottya Júlia Ujfalussy[1,2,5*], Zsófia Bognár[1,2], Marianna Molnár[3], Ádám Miklósi[1], Enikő Kubinyi[1,2,4]

1 Department of Ethology, Institute of Biology, Eötvös Loránd University, Budapest, Hungary

2 MTA-ELTE Lendület "Momentum" Companion Animal Research Group, Budapest, Hungary

3 Centre for Environmental Research, Faculty of Natural Sciences, Eötvös Loránd University, Budapest, Hungary

4 ELTE NAP Canine Brain Research Group

5 Institute of Cognitive Neuroscience and Psychology, Research Centre for Natural Sciences, Budapest, Hungary

*Corresponding author:

Dorottya Júlia Ujfalussy

Department of Ethology, ELTE Eötvös Loránd University, Budapest

Pázmány Péter sétány 1/C, H-1117, Hungary.

E-Mail: ujfalussydori@gmail.com

English Bulldog **EB**

**BOX A**		Proportion of total time					Sec
	Name	OrientOwner	OrientExp	OrientBox	PawUse	NoseUse	OpenLat
**1.**	**Bowling**	36,5	16,83	13,83	0	9	120
**2.**	**Archibald**	0	5,88	94,12	0	94,12	3,4
**3.**	**Shiva**	23,03	33,28	11,93	1,18	9,58	120
**4.**	**Bosco**	21,75	25,99	20,42	0	18,3	75,4
**5.**	**Rocco**	17,11	17,29	1,5	0	0,94	120
**6.**	**Hektor**	6,24	23,02	18,94	0	18,23	83,4
**7.**	**Jack**	16,64	38,03	3,9	0	1,36	120
**8.**	**Fotel**	16,55	15,7	5,29	0	3,75	120
**9.**	**Lamantin**	7,83	40	37,83	0	29,67	120
**10.**	**Brisz**	0	0	100	0	100	2
**11.**	**Dagi**	31,13	0	60,93	0	59,6	30,2
**12.**	**Wellington**	18,46	33,85	47,69	0	33,85	13
**13.**	**Gyömösz**	0	20	80	0	60	3
**14.**	**Fióna**	22,83	45,17	18,17	0,83	16,67	120
**15.**	**Szamóca**	0	35,71	64,29	0	45,24	16,8

**BOX B**		Proportion of total time					Sec
	Name	OrientOwner	OrientExp	OrientBox	PawUse	NoseUse	OpenLat
**1.**	**Bowling**	10,08	11,14	45,89	1,06	37,67	75,4
**2.**	**Archibald**	13,67	15,83	56,33	0	52,67	120
**3.**	**Shiva**	15,64	16,2	55,87	0	40,22	35,8
**4.**	**Bosco**	12,78	15,04	25,56	3,76	15,79	26,6
**5.**	**Rocco**	26,84	15,04	37,46	0	35,99	67,8
**6.**	**Hektor**	0	0	100	0	76,92	2,6
**7.**	**Jack**	21,85	15,97	22,69	2,52	21,01	23,8
**8.**	**Fotel**	7,91	31,07	24,11	1,13	22,03	120
**9.**	**Lamantin**	4,17	45,33	8,33	0	4,83	120
**10.**	**Brisz**	0,65	14,98	71,01	3,91	65,47	61,4
**11.**	**Dagi**	17,11	18,77	21,1	2,33	14,29	120
**12.**	**Wellington**	6,8	44,9	11,81	0	7,51	120
**13.**	**Gyömösz**	32,6	20,71	30,05	3,23	20,2	120
**14.**	**Fióna**	13,33	25,67	44	0,67	43	120
**15.**	**Szamóca**	8,8	24,72	58,99	0	56,55	106,8

**BOX C**		Proportion of total time					Sec
	Name	OrientOwner	OrientExp	OrientBox	PawUse	NoseUse	OpenLat
**1.**	**Bowling**	0	5,17	94,83	24,14	43,1	11,6
**2.**	**Archibald**	0	0	100	0	92,31	2,6
**3.**	**Shiva**	0	0	100	0	100	1,2
**4.**	**Bosco**	0	0	64,71	0	35,29	3,4
**5.**	**Rocco**	14,07	19,93	6,53	0	5,03	120
**6.**	**Hektor**	0	36,36	63,64	0	63,64	2,2
**7.**	**Jack**	12,42	43,21	1,99	0	1,99	120
**8.**	**Fotel**	0	38,89	61,11	0	47,22	7,2
**9.**	**Lamantin**	0	43,51	39,69	0	34,35	52,4
**10.**	**Brisz**	0	0	100	0	92,59	5,4
**11.**	**Dagi**	0	0	100	0	100	2,8
**12.**	**Wellington**	0	50	50	0	43,48	9,2

**13.**	**Gyömösz**	0	3,85	96,15	11,54	61,54	5,2
**14.**	**Fióna**	0	32,84	55,22	0	25,37	13,4
**15.**	**Szamóca**	0	0	100	0	100	2

French Bulldog **FB**

**BOX A**		Proportion of total time					Sec
	Name	OrientOwner	OrientExp	OrientBox	PawUse	NoseUse	OpenLat
**1.**	**Joey**	24,16	29,78	41,57	0	41,01	35,6
**2.**	**Lola**	18,2	29,46	23,05	0	19,41	120
**3.**	**Luna**	7,89	28,99	44,6	0	39,11	120
**4.**	**Buffy**	2,19	34,31	59,12	0	56,93	27,4
**5.**	**Ottó**	0	0	100	0	100	3
**6.**	**Pepe**	11,6	35,15	14,24	0	10,37	120
**7.**	**Molly**	5,63	19,8	46,08	2,56	42,49	120
**8.**	**Lunaa**	30,12	0	57,83	0	54,22	16,6
**9.**	**Zoé**	15,33	28,22	28,44	0	26,44	90
**10.**	**Mása**	11,18	22,37	61,84	0	60,53	30,4
**11.**	**Villám**	0	0	100	0	100	9,4
**12.**	**Gabriella**	5,8	16,67	13,77	0	10,87	120
**13.**	**Szandi**	0	0	94,12	11,76	80	17
**14.**	**Dior**	0	0	100	0	100	5,2
**15.**	**Jade**	5,99	45,21	39,82	8,38	29,64	66,8

**BOX B**		Proportion of total time					Sec
	Name	OrientOwner	OrientExp	OrientBox	PawUse	NoseUse	OpenLat
**1.**	**Joey**	18,33	39,5	35,5	1	33,83	120
**2.**	**Lola**	36,09	11,13	31,53	0	30,19	120
**3.**	**Luna**	10,98	38,51	21,28	0	13,85	120
**4.**	**Buffy**	15,66	27,44	39,9	0	38,72	120
**5.**	**Ottó**	8,14	13,29	4,65	0	4,15	120
**6.**	**Pepe**	8,76	23,88	9,45	0	8,42	120
**7.**	**Molly**	0	0	100	0	95,74	9,4
**8.**	**Lunaa**	22	27,17	3,17	0	2,67	120
**9.**	**Zoé**	16,41	18,97	26,5	0	25,47	120
**10.**	**Mása**	12,17	37,74	46,56	3,35	41,45	120
**11.**	**Villám**	1,61	10,14	46,59	5,73	40,32	120
**12.**	**Gabriella**	6,77	41,04	26,69	0	23,51	50,2
**13.**	**Szandi**	11,98	0	77,84	4,79	66,47	33,4
**14.**	**Dior**	0	20,67	50,67	7,33	41,5	120
**15.**	**Jade**	0	11,76	85,88	0	81,18	34

**BOX C**		Proportion of total time					Sec
	Name	OrientOwner	OrientExp	OrientBox	PawUse	NoseUse	OpenLat
**1.**	**Joey**	25,49	53,43	14,22	0	12,25	40,8
**2.**	**Lola**	29,81	41,35	15,38	0	4,81	20,8
**3.**	**Luna**	23,26	2,33	65,12	0	46,51	8,6
**4.**	**Buffy**	0	29,23	58,46	0	53,85	13
**5.**	**Ottó**	0	0	100	0	100	9,6
**6.**	**Pepe**	0	11,11	88,89	0	88,89	3,6
**7.**	**Molly**	0	0	100	0	75	0,8
**8.**	**Lunaa**	0	0	100	0	100	0,4
**9.**	**Zoé**	22,17	9,91	10,85	0	10,38	42,4

**10.**	**Mása**	8	51,76	25,65	0	25,18	85
**11.**	**Villám**	0	47,22	41,67	0	30,56	14,4
**12.**	**Gabriella**	0	47,62	38,1	0	28,57	8,4
**13.**	**Szandi**	4,65	20,93	60,47	0	55,81	17,2
**14.**	**Dior**	0	2,23	88,85	0	86,99	53,8
**15.**	**Jade**	0	11,11	88,89	0	88,89	1,8

Mudi **M**

BOX A		Proportion of total time					Sec
	Name	OrientOwner	OrientExp	OrientBox	PawUse	NoseUse	OpenLat
**1.**	**Fruti**	7,26	7,66	35,89	10,08	0	49,4
**2.**	**Bojtár**	0	0	79,41	0	44,12	6,6
**3.**	**Kedves**	0	33,17	30,15	0	17,59	39,6
**4.**	**Lepke**	0	0	85,71	0	28,57	1,2
**5.**	**Pletyka**	0	0	96	0	76	4,8
**6.**	**Csinos**	20,48	28,06	15,16	2,74	6,29	120
**7.**	**Tipli**	0	0	97,37	84,21	10,53	7,4
**8.**	**Villám**	0	5,65	74,19	0	34,68	24,6
**9.**	**Ágas**	0	10	90	15	65	3,8
**10.**	**Akác**	8,45	14,59	26,3	0	13,63	120
**11.**	**Alfie**	0	0	100	0	66,67	6,4
**12.**	**Bikfic**	0	4,55	95,45	63,64	0	37,8
**13.**	**Bodor**	0	0	100	12	68	4,8
**14.**	**Ében**	0	0	100	21,43	64,29	2,6
**15.**	**Csele**	4,95	3,3	53,47	16,5	31,68	60,4

BOX B		Proportion of total time					Sec
	Name	OrientOwner	OrientExp	OrientBox	PawUse	NoseUse	OpenLat
**1.**	**Fruti**	0	0	98,82	0	92,94	16,8
**2.**	**Bojtár**	0	0	91,67	0	71,88	19
**3.**	**Kedves**	0	0	97,67	14,73	82,95	25,6
**4.**	**Lepke**	0	7,24	66,21	2,41	37,93	57,8
**5.**	**Pletyka**	0	0	89,91	68,81	20,64	43,4
**6.**	**Csinos**	0	0	100	18,75	75	9,4
**7.**	**Tipli**	0	0	76,3	31,21	52,6	34,4
**8.**	**Villám**	0	0	100	69,57	0	4,4
**9.**	**Ágas**	0	5,63	65,8	12,99	45,89	46
**10.**	**Akác**	14,94	6,33	31,17	22,73	0,49	120
**11.**	**Alfie**	0	0	100	82,54	0	12,4
**12.**	**Bikfic**	2,63	0	48,95	1,58	23,16	4,2
**13.**	**Bodor**	0	0	100	83,33	18,75	9,4
**14.**	**Ében**	0	6,52	45,65	2,9	31,88	27,4
**15.**	**Csele**	0	0	100	14,71	73,53	6,6

BOX C		Proportion of total time					Sec
	Name	OrientOwner	OrientExp	OrientBox	PawUse	NoseUse	OpenLat
**1.**	**Fruti**	0	0	100	0	50	2,2
**2.**	**Bojtár**	4,32	3,78	57,84	1,62	32,43	36,8
**3.**	**Kedves**	0	0	100	75	8,33	2,2
**4.**	**Lepke**	0	0	94,12	47,06	5,88	3,2
**5.**	**Pletyka**	0	0	85,25	0	59,02	24,2
**6.**	**Csinos**	0	10,83	71,67	0	55,83	23,8

**7.**	**Tipli**	0	0	93,33	0	40	2,8
**8.**	**Villám**	0	0	75	0	25	1,4
**9.**	**Ágas**	0	7,14	85,71	42,86	0	2,6
**10.**	**Akác**	22,36	12,66	25,74	0	9,28	47,2
**11.**	**Alfie**	0	0	100	0	52,38	4
**12.**	**Bikfic**	18,52	19,75	24,69	0	12,35	32,2
**13.**	**Bodor**	0	8,45	50,7	0	33,8	14
**14.**	**Ében**	0	0	100	31,58	47,37	3,6
**15.**	**Csele**	0	0	93,33	0	63,33	5,8
